# Prognostic significance of plasma SDF-1 in acute ischemic stroke patients with diabetes mellitus: the CATIS trial

**DOI:** 10.1186/s12933-023-01996-0

**Published:** 2023-10-10

**Authors:** Shoujiang You, Hongyu Chen, Mengyuan Miao, Jigang Du, Bizhong Che, Tan Xu, Chun-Feng Liu, Yonghong Zhang, Jiang He, Xiaoyan Zhong, Yongjun Cao, Chongke Zhong

**Affiliations:** 1 Department of Neurology and Suzhou Clinical Research Center of Neurological Disease, The Second Affiliated Hospital of Soochow University, No.1055, Sanxiang Road, Suzhou, 215004 China; 2 Department of Epidemiology, School of Public Health, Jiangsu Key Laboratory of Preventive and Translational Medicine for Geriatric Diseases, MOE Key Laboratory of Geriatric Diseases and Immunology, Suzhou Medical College of Soochow University, 199 Renai Road, Industrial Park District, Suzhou, 215123 China; 3 Institutes of Neuroscience, Soochow University, Suzhou, 215123 China; 4 Department of Epidemiology, Tulane University School of Public Health and Tropical Medicine, New Orleans, LA USA; 5 School of Public Health, Suzhou Medical College of Soochow University, 199 Renai Road, Industrial Park District, Suzhou, Jiangsu 215123 China

**Keywords:** Acute ischemic stroke, Stromal cell-derived factor-1, Prognosis, Diabetes mellitus

## Abstract

**Background and objectives:**

Evidence on the associations between baseline stromal cell-derived factor (SDF)-1 and clinical outcomes in acute ischemic stroke patients is lacking. The present study aimed to examine the relationship between plasma SDF-1 levels and clinical outcomes based on a large multicenter study of the China Antihypertensive Trial in Acute Ischemic Stroke (CATIS).

**Methods:**

Secondary analysis was conducted among 3,255 participants from the CATIS trial with a baseline measurement of plasma SDF-1 levels. We evaluated the associations between plasma SDF-1 levels and one-year recurrent stroke, cardiovascular events, and all-cause mortality using Cox regression models. We further investigated the prognostic effect of SDF-1 on clinical outcomes in patients with different characteristics.

**Results:**

Higher plasma SDF-1 levels were not associated with recurrent stroke, cardiovascular events, and all-cause mortality at one-year after ischemic stroke (all *P* trend ≥ 0.05). There were significant interactions between plasma SDF-1 levels and history of diabetes mellitus on recurrent stroke (*P* = 0.005), cardiovascular events (*P* = 0.007) and all-cause mortality (*P* = 0.04) at one year. In patients with diabetes mellitus, plasma SDF-1 was significantly associated with an increased risk of recurrent stroke and cardiovascular events after adjustment for confounders. For example, 1-SD higher log–SDF-1 was associated with a hazard ratio (95% confidence interval) of 1.65 (1.18–2.32) for recurrent stroke and 1.47 (1.08–1.99) for the cardiovascular events, but not all-cause mortality 1.36 (0.96–1.93) at one year. However, there were no associations between plasma SDF-1 and clinical outcomes in patients without diabetes mellitus (all *P* > 0.05). The addition of plasma SDF-1 to the conventional risk factors model significantly improved the risk prediction of all outcomes. Similarly, findings between elevated SDF-1 levels and two-year outcomes were found only in patients with diabetes mellitus.

**Conclusions:**

Elevated plasma SDF-1 was significantly associated with an increased risk of recurrent stroke and cardiovascular events only in ischemic patients with diabetes mellitus.

**Supplementary Information:**

The online version contains supplementary material available at 10.1186/s12933-023-01996-0.

## Introduction

Identifying novel biomarkers of recurrent stroke and cardiovascular events after stroke could increase our understanding of its pathogenesis and suggest novel therapeutic targets. The stromal cell-derived factor (SDF)-1, also known as CXC chemokine ligand-12 (CXCL-12), is a member of the CXC chemokine subfamily and regulates cellular activity by binding to CXC chemokine receptor 4 (CXCR4) or CXCR7 [1–3].

Experimental studies indicated that increased SDF-1 could induce neointimal hyperplasia and angiogenesis, triggers vascular inflammatory response, aggravates insulin resistance, and is associated with atherosclerosis and cardiovascular events [1–7]. Several population studies have proven the positive association of serum SDF-1 with the risk of cardiovascular disease, including ischemic stroke [1, 4–7]. Moreover, some studies also indicated that a high level of serum SDF-1 was associated with an increased risk of poor functional outcome and animal studies reported that the SDF-1 antagonist AMD3100 significantly improved functional recovery after stroke [8–10]. However, epidemiological evidence with sufficient statistical power on the association between serum SDF-1 and recurrent stroke and cardiovascular events after acute ischemic stroke is limited [11].

Interestingly, several studies have noted a strong association between serum SDF-1 and diabetes mellitus [12–15]. These studies demonstrated that the levels of serum SDF-1 were increased in patients with diabetes mellitus, and increased SDF-1 can aggravate insulin resistance, and promote chronic inflammation and immune responses, which are the main mechanisms of diabetes mellitus progression and diabetes-associated cardiovascular events [12–15].

We hypothesized that having higher level of SDF-1 may be associated with an increased risk of recurrent stroke and cardiovascular events after ischemic stroke and the effect of SDF-1 on ischemic stroke prognosis may be different in patients with and without diabetes mellitus. To test this hypothesis, we conducted a prospective study to investigate the association between plasma SDF-1 levels and recurrent stroke, cardiovascular events, and all-cause mortality in ischemic stroke patients based on a large prospective multicenter study of the China Antihypertensive Trial in Acute Ischemic Stroke (CATIS).

## Methods

### Study participants

The present study is a secondary analysis of the CATIS trial, a multicenter, single-blind, blinded endpoints, randomized clinical trial conducted in 26 hospitals across China from August 2009 to May 2013, and the purpose of CATIS was to evaluate whether immediate blood pressure (BP) reduction in patients with acute ischemic stroke could reduce death and major disability at 14 days or hospital discharge [16]. The CATIS trial was registered on clinicaltrials.gov (NCT01840072). Details of the rationale, design, and major results of CATIS had been reported previously. Briefly, 4071 patients aged ≥ 22 years with a first-ever ischemic stroke, confirmed by computed tomography or magnetic resonance imaging of the brain within 48 h of symptom onset, and with elevated systolic blood pressure (BP) between 140 to < 220 mmHg were recruited. Patients with a BP ≥ 220/120 mmHg, severe heart failure, acute myocardial infarction or unstable angina, atrial fibrillation, aortic dissection, severe cerebrovascular stenosis (≥ 70%), or resistant hypertension, in a deep coma, or treated with intravenous thrombolytic therapy were excluded from the CATIS trial [16]. Patients were randomly assigned to receive antihypertensive treatment (aimed at lowering systolic blood pressure by 10–25% within the first 24 h after randomization, achieving blood pressure less than 140/90 mm Hg within seven days, and maintaining this level during hospitalization) or to discontinue all antihypertensive medications (control) during hospitalization.

The current study excluded 632 patients without available blood samples for plasma SDF-1 measurements and 184 patients lost to follow-up. Finally, 3,255 ischemic stroke patients from CATIS were included for analysis (Supplementary Fig. 1).

### Data collection

Baseline data on demographic characteristics, lifestyle risk factors, medical history, and medication use history were collected upon hospital admission using a standard questionnaire. Information on baseline characteristics was obtained through face-to-face interviews with trained interviewers. Stroke severity was assessed using the National Institutes of Health Stroke Scale (NIHSS) by trained neurologists at admission. Ischemic stroke was classified as large-artery atherosclerosis (embolus/thrombosis), cardiac embolism (embolic), and small-artery occlusion (lacunar) according to the symptoms and imaging data of the patients. Three blood pressure (BP) measurements were also obtained at admission by trained nurses using a standard mercury sphygmomanometer according to a standard protocol adapted from procedures recommended by the American Heart Association [17]. Additionally, serum lipids, plasma glucose, and other clinical laboratory measurements were obtained at the participating hospitals at admission.

Blood samples were collected after at least eight hours of fasting within 24 h of hospital admission. Plasma samples were separated at the clinical laboratories of the participating hospitals and immediately frozen at − 80 °C. Plasma SDF-1 concentrations were measured centrally in the Central Laboratory of the School of Public Health at Soochow University with a commercially available ELISA kit (Catalog: DY350; R&D Systems, Minneapolis, Minnesota). Intra- and inter-assay coefficients of variation were 5.8% and 8.6%, respectively. Laboratory technicians who measured plasma SDF-1 levels were blinded to baseline characteristics and study clinical outcomes of participants.

### Study outcomes

Participants were followed up in person two years after stroke onset by trained neurologists and research nurses blinded to treatment assignment. The primary outcome was a recurrent stroke one year after stroke onset. The secondary outcomes were: (1) composite of cardiovascular events after ischemic stroke, including vascular death, nonfatal stroke, nonfatal myocardial infarction, angina hospitalization, congestive heart failure hospitalization, peripheral arterial disease at one year; (2) all-cause mortality at one year after stroke onset. Death certificates were obtained for deceased participants, and hospital data were abstracted for all vascular events. The study outcomes assessment committee blinded to treatment assignment reviewed and adjudicated outcomes based on the criteria established in the Antihypertensive and Lipid-Lowering Treatment to Prevent Heart Attack Trial.

### Statistical analysis

We grouped all study participants by quartiles of plasma SDF-1 levels. Cox proportional hazards models were used to assess the associations between plasma SDF-1 and recurrent stroke, cardiovascular events, and all-cause mortality in all study participants and key subgroups by age (≥ 62 vs. <62 years), sex, current smoking, alcohol consumption, baseline NIHSS score, history of hypertension, randomization treatment and history of diabetes mellitus. The interaction between subgroup valuables and SDF-1 on study outcome was tested by the likelihood ratio test of models with interaction terms, adjusting for the covariates.

In the subgroup analysis, we found significant interactions between plasma SDF-1 levels and the status of diabetes mellitus on the study outcomes at one year. Then, the participants were divided into two subgroups according to diabetes mellitus status. In each subgroup, the participants were further divided into four groups by quartiles of plasma SDF-1 levels. Baseline characteristics were compared and tested for trends across the SDF-1 quartiles using a generalized linear model in patients with and without diabetes mellitus. The associations between plasma SDF-1 and recurrent stroke, cardiovascular events, and all-cause mortality were assessed using Cox proportional hazards models in patients with and without diabetes. The covariates included in the multivariable models were selected based on prior knowledge, including age, sex, current smoking, alcohol consumption, estimated glomerular filtration rate (eGFR), baseline NIHSS score, medical history (hypertension, hyperlipidemia), ischemic stroke subtype, and randomized treatment. The proportional hazards assumption of the Cox models was tested using Schoenfeld residuals, which showed no significant departure from proportionality (*P* > 0.05). Hazard ratios (HRs) and 95% confidence intervals (CIs) for SDF-1 quartiles and per one standard deviation (SD) increment of log-transformed plasma SDF-1 levels were calculated. Tests for linear trends in HRs across plasma SDF-1 quartiles were conducted using the median value of each quartile as the predictor. In addition, restricted cubic splines were used to generate more precise estimates and to assess the shape of associations between SDF-1 and clinical outcomes (with four knots at the 5th, 35th, 65th, and 95th percentiles) [18]. The Fine-Gray model was performed as a sensitivity analysis to robustly assess the association between SDF-1 levels and recurrent stroke and cardiovascular events (excluding vascular death) by regarding death as a competing risk [19].

We further assessed the predictive performance of plasma SDF-1 for clinical outcomes in the basic model with conventional risk factors. The Hosmer Lemeshow χ^2^ statistic was first conducted to evaluate the calibration of models with SDF-1. Furthermore, the net reclassification index (NRI) and integrated discrimination improvement (IDI) were used for assessing improvements in risk reclassification when adding plasma SDF-1 to the conventional model [20, 21]. Multiple imputations for missing covariate values were performed using the Markov chain Monte Carlo method. To assess the robustness of the predicted effect of plasma SDF-1 after stroke, we also analyzed the association between plasma SDF-1 levels and recurrent stroke, cardiovascular events, and all-cause mortality at two years as sensitivity analysis. All *P* values were 2-tailed, and a significance level of 0.05 was used. Statistical analysis was conducted using SAS statistical software (version 9.2, Cary, North Carolina, USA).

## Results

### Baseline characteristics

A total of 3,255 ischemic stroke patients (mean [SD] age 61.9 [10.8] years, 2103 [64.6%] male) were included in the present analyses. The median plasma SDF-1 concentration was 639.4 pg/mL (interquartile range 436.9-855.3 pg/mL). The level of plasma SDF-1 in patients with diabetes mellitus was lower than in those without diabetes mellitus (624.5 pg/mL vs. 673.7 pg/mL). The baseline characteristics of participants with and without diabetes mellitus are shown in Supplementary Table 1. Compared with participants without diabetes mellitus, patients with diabetes mellitus were less often male, smokers, and drinkers, had lower diastolic BP, higher levels of triglycerides, total cholesterol, low-density lipoprotein cholesterol and fasting plasma glucose and lower levels of high-density lipoprotein cholesterol. Patients with diabetes mellitus also had a greater frequency of history of hypertension, hyperlipidemia, and coronary heart disease, prior antihypertensive and lipid-lowing treatment. The frequency of recurrent stroke, cardiovascular events, and all-cause mortality in patients with diabetes mellitus was higher than those without diabetes mellitus, although no statistically significant difference was observed between the two groups (Supplementary Table 2).

Among 567 patients with diabetes mellitus, compared with participants with lower plasma SDF-1 levels, those with higher SDF-1 were older, had lower levels of eGFR, more likely with lacunar stroke, and less likely with thrombosis (Table 1). In 2688 patients without diabetes mellitus, compared with participants with lower plasma SDF-1 levels, those with higher SDF-1 were older and female, non-smoking, non-alcohol drinking, and more likely to have a lacunar stroke and less likely to have thrombosis. Patients with higher SDF-1 levels also had lower levels of total cholesterol, eGFR and a longer time from onset to hospitalization (Table 1).

**Table 1:** Baseline characteristics of the study participants by diabetes status according to SDF-1 quartiles

		Ischemic stroke patients with diabetes					Ischemic stroke patients without diabetes			
**Characteristics**	**Q1**	**Q2**	**Q3**	**Q4**	***P***_trend_	**Q1**	**Q2**	**Q3**	**Q4**	***P***_trend_
No. of participants	166	148	126	127		646	668	687	687	
**Demographics**										
Age, years	60.2 ± 9.6	60.9 ± 9.5	63.1 ± 9.3	63.6 ± 9.8	0.0006	60.5 ± 11.4	60.6 ± 11.2	62.3 ± 10.4	64.1 ± 11.0	< 0.0001
Male	96 (57.8)	86 (58.1)	84 (66.7)	72 (56.7)	0.74	440 (68.1)	464 (69.5)	427 (62.2)	434 (63.2)	0.007
Current cigarette smoking	56 (33.7)	37 (25.0)	40 (31.8)	32 (25.2)	0.24	276 (42.7)	253 (37.9)	248 (36.1)	257 (37.4)	0.04
Current alcohol drinking	45 (27.1)	36 (24.3)	23 (18.3)	35 (27.6)	0.70	232 (35.9)	249 (37.3)	206 (30.0)	195 (28.4)	0.0002
**Clinical features**										
SBP, mm Hg	164.2 ± 16.3	166.5 ± 16.8	164.2 ± 17.4	167.0 ± 17.5	0.33	166.9 ± 16.7	166.5 ± 16.9	166.3 ± 16.8	166.2 ± 16.6	0.45
DBP, mm Hg	94.2 ± 9.9	95.1 ± 11.6	94.7 ± 9.9	96.8 ± 12.3	0.08	97.5 ± 11.1	97.0 ± 10.9	97.4 ± 11.5	96.2 ± 10.8	0.08
WBC, 10^9^/L	7.9 ± 5.6	7.5 ± 3.4	7.7 ± 4.0	7.7 ± 2.9	0.70	8.8 ± 33.5	7.9 ± 5.2	8.3 ± 27.6	7.3 ± 3.1	0.28
TC, mmol/L	5.3 (4.3–6.1)	5.1 (4.3–5.8)	5.0 (4.4–5.7)	5.2 (4.3-6.0)	0.26	5.1 (4.4–5.8)	4.9 (4.3–5.6)	4.9 (4.3–5.7)	4.8 (4.1–5.7)	0.02
TG, mmol/L	1.7 (1.2–2.6)	1.6 (1.2–2.5)	1.8 (1.3–2.5)	2.0 (1.3–2.8)	0.93	1.5 (1.0-2.2)	1.4 (1.0-2.1)	1.5 (1.0-2.2)	1.4 (1.0–2.0)	0.76
LDL-C, mmol/L	3.0 (2.2–3.7)	3.1 (2.3–3.6)	3.0 (2.4–3.6)	3.0 (2.4–3.6)	0.78	2.9 (2.3–3.6)	2.8 (2.3–3.4)	2.8 (2.3–3.5)	2.8 (2.2–3.5)	0.23
HDL-C, mmol/L	1.1 (0.9–1.4)	1.2 (1.0-1.4)	1.2 (1.0-1.4)	1.2 (1.0-1.4)	0.74	1.3 (1.0-1.5)	1.2 (1.0-1.5)	1.3 (1.0-1.5)	1.3 (1.0-1.6)	0.08
Time from onset to hospitalization, h	10.0 (5.0–24.0)	12.0 (5.0–24.0)	11.0 (4.5–24.0)	10.0 (4.0–24.0)	0.78	9.0 (4.0–24.0)	10.0 (5.0–24.0)	11.5 (4.4–24.0)	12.0 (4.5–24.0)	0.0002
FPG, mmol/L	9.6 ± 3.9	9.1 ± 3.1	9.1 ± 3.8	9.5 ± 4.0	0.79	6.2 ± 2.4	6.0 ± 1.9	6.2 ± 2.1	6.0 ± 1.8	0.24
eGFR, ml/min/1.73 m2	109.6 (97.2-119.2)	106.3 (95.8-115.5)	103.3 (92.8-111.2)	101.9 (84.6-111.1)	0.0005	108.2 (97.6-117.6)	106.7 (96.5-116.1)	103.6 (92.6-112.3)	100.9 (87.0-111.1)	< 0.0001
Baseline NIHSS score	4.0 (2.0–7.0)	4.0 (3.0–7.0)	4.0 (3.0–8.0)	4.0 (3.0–8.0)	0.44	4.0 (2.0–7.0)	5.0 (3.0–8.0)	5.0 (3.0–8.0)	5.0 (2.0–8.0)	0.42
**Medical history**										
Hypertension	137 (82.5)	129 (87.2)	109 (86.5)	111 (87.4)	0.26	495 (76.6)	518 (77.5)	533 (77.6)	523 (76.1)	0.83
Hyperlipidemia	24 (14.5)	27 (18.2)	19 (15.1)	20 (15.8)	0.92	27 (4.2)	33 (4.9)	50 (7.3)	34 (5.0)	0.24
Coronary heart disease	19 (11.5)	31 (21.0)	21 (16.7)	25 (19.7)	0.12	50 (7.7)	54 (8.1)	70 (10.2)	64 (9.3)	0.17
Family history of stroke	47 (28.3)	26 (17.6)	22 (17.5)	25 (19.7)	0.06	131 (20.3)	109 (16.3)	108 (15.7)	134 (19.5)	0.69
**Prior use of medications**										
Antihypertensive	98 (59.0)	94 (63.5)	74 (58.7)	73 (57.5)	0.65	313 (48.5)	304 (45.5)	317 (46.1)	304 (44.3)	0.17
Lipid-lowing	12 (7.2)	8 (5.4)	8 (6.4)	11 (8.7)	0.63	9 (1.4)	17 (2.5)	22 (3.2)	20 (2.9)	0.06
**Ischemic stroke subtype**										
Thrombotic	140 (84.3)	115 (77.7)	94 (74.6)	91 (71.7)	0.008	516 (79.9)	522 (78.1)	538 (78.3)	491 (71.5)	0.0008
Embolic	5 (3.0)	9 (6.1)	6 (4.8)	3 (2.4)	0.97	29 (4.5)	36 (5.4)	35 (5.1)	29 (4.2)	0.72
Lacunar	21 (12.7)	24 (16.2)	26 (20.6)	33 (26.0)	0.002	101 (15.6)	110 (16.5)	114 (16.6)	167 (24.3)	< 0.0001
Randomized treatment	92 (55.4)	75 (50.7)	56 (44.4)	66 (52.0)	0.33	312 (48.3)	331 (49.6)	355 (51.7)	350 (51.0)	0.25

Cut-points: Q1: < 436.9; Q2: 436.9-639.4; Q3: 639.4-855.3; Q4:≥855.3

Continuous variables are expressed as mean ± standard deviation or median (interquartile range). Categorical variables are expressed as frequency (percentage)

Abbreviations: SDF-1, stromal cell-derived factor-1; FPG, fasting plasma glucose; NIHSS, National Institute of Health Stroke Scale; SBP, systolic blood pressure; DBP, diastolic blood pressure; TG, triglycerides; TC, total cholesterol; LDL-C, low-density lipoprotein cholesterol; HDL-C, high-density lipoprotein cholesterol; WBC, white blood cell; eGFR, estimated glomerular filtration rate

### Plasma SDF-1 levels and clinical outcomes after acute ischemic stroke

During one year of follow-up, there were 150 recurrent strokes (4.6%), 196 cardiovascular events (6.0%), and 204 all-cause mortalities (6.3%) in the whole population. We found that plasma SDF-1 levels were not associated with recurrent stroke, cardiovascular events, and all-cause mortality one year after stroke onset Supplementary Table 3). There were significant interactions between plasma SDF-1 levels and history of diabetes mellitus on recurrent stroke (*P* = 0.005 for interaction), cardiovascular events (*P* = 0.007 for interaction) and, all-cause mortality (*P* = 0.04 for interaction) at one year. However, no significant interaction between plasm SDF-1 and age, sex, current smoking, alcohol consumption, baseline NIHSS score, history of hypertension, and randomization treatment on outcomes were observed (all *P* for interaction > 0.05).

In 567 patients with diabetes mellitus, during one year of follow-up, there were 30 recurrent strokes (5.3%), 42 cardiovascular events (7.4%), and 40 all-cause mortalities (7.1%). Compared to those with the lowest quartile of plasma SDF-1 levels, patients with the highest SDF-1 levels had a higher risk HRs (95% CIs) of recurrent stroke [4.12 (1.31–12.95)], cardiovascular events [2.80 (1.11–7.06)] but not all-cause mortality [2.09 (0.86–5.09)] at one year after adjustment for age, sex, medical history, and other covariates (Table 2). Each 1-SD increase in log-transformed SDF-1 levels was associated with a 65% increased risk (HR 1.65, 95% CI 1.18–2.32) of recurrent stroke and a 47% increased risk (HR 1.47, 95% CI 1.08–1.99) of cardiovascular events at one year after stroke (Table 2). There were 120 recurrent strokes (4.5%), 154 cardiovascular events (5.7%), and 164 all-cause mortalities (6.1%) at one year in non-diabetic patients. Patients with the highest SDF-1 levels did not have an increased risk of recurrent stroke [0.95 (0.58–1.55)], cardiovascular events [0.71 (0.45–1.12)] and all-cause mortality [0.84 (0.53–1.33)] at one year in compared to those with the lowest quartile of plasma SDF-1 levels (Table 2). In a competing risk analysis, the associations of recurrent stroke and cardiovascular events (excluding vascular death) with increased SDF-1 levels in patients with diabetes mellitus at one year were still significant treating death as a competing risk (Supplementary Table 4).

**Table 2:** Associations between SDF-1 quartiles and stroke outcomes according to diabetes status

	SDF-1, pg/mL					Each 1-SD increase	
	**Q1**	**Q2**	**Q3**	**Q4**	***P *** **trend**		
**Ischemic stroke patients with diabetes**							
**Recurrent stroke at 1 year**							
Events, n (%)	4 (2.4)	6 (4.1)	8 (6.4)	12 (9.5)			
Model 1	1.00	1.72 (0.49–6.10)	2.60 (0.78–8.68)	4.00 (1.29–12.41)	0.008	1.67 (1.18–2.35)	
Model 2	1.00	1.79 (0.51–6.35)	2.65 (0.79–8.89)	4.16 (1.34–12.94)	0.007	1.65 (1.18–2.31)	
Model 3	1.00	1.75 (0.49–6.21)	2.76 (0.81–9.38)	4.12 (1.31–12.95)	0.007	1.65 (1.18–2.32)	
**Cardiovascular events at 1 year**							
Events, n (%)	7 (4.2)	9 (6.1)	12 (9.5)	14 (11.0)			
Model 1	1.00	1.48 (0.55–3.98)	2.18 (0.86–5.57)	2.64 (1.06–6.54)	0.02	1.43 (1.06–1.93)	
Model 2	1.00	1.51 (0.56–4.06)	2.21 (0.86–5.64)	2.68 (1.08–6.66)	0.02	1.43 (1.06–1.93)	
Model 3	1.00	1.46 (0.54–3.92)	2.23 (0.86–5.79)	2.80 (1.11–7.06)	0.02	1.47 (1.08–1.99)	
**All-cause mortality at 1 year**							
Events, n (%)	9 (5.4)	8 (5.4)	9 (7.1)	14 (11.0)			
Model 1	1.00	1.02 (0.39–2.65)	1.28 (0.51–3.25)	1.94 (0.84–4.48)	0.09	1.27 (0.92–1.74)	
Model 2	1.00	1.00 (0.39–2.60)	1.27 (0.50–3.22)	1.89 (0.82–4.39)	0.10	1.26 (0.91–1.73)	
Model 3	1.00	0.90 (0.35–2.37)	1.36 (0.53–3.53)	2.09 (0.86–5.09)	0.07	1.36 (0.96–1.93)	
**Ischemic stroke patients without diabetes**							
**Recurrent Stroke at 1 year**							
Events, n (%)	33 (5.1)	29 (4.3)	25 (3.6)	33 (4.8)			
Model 1	1.00	0.85 (0.52–1.40)	0.69 (0.41–1.16)	0.90 (0.56–1.47)	0.61	0.92 (0.76–1.11)	
Model 2	1.00	0.85 (0.52–1.41)	0.70 (0.42–1.18)	0.91 (0.56–1.48)	0.63	0.92 (0.77–1.11)	
Model 3	1.00	0.85 (0.52–1.40)	0.70 (0.42–1.18)	0.95 (0.58–1.55)	0.77	0.94 (0.78–1.13)	
**Cardiovascular events at 1 year**							
Events, n (%)	43 (6.7)	38 (5.7)	38 (5.5)	35 (5.1)			
Model 1	1.00	0.86 (0.55–1.32)	0.80 (0.51–1.23)	0.72 (0.46–1.13)	0.15	0.88 (0.74–1.04)	
Model 2	1.00	0.49 (0.55–1.33)	0.80 (0.52–1.25)	0.73 (0.46–1.14)	0.16	0.88 (0.74–1.04)	
Model 3	1.00	0.83 (0.54–1.29)	0.79 (0.51–1.22)	0.71 (0.45–1.12)	0.14	0.87 (0.74–1.04)	
**All-cause mortality at 1 year**							
Events, n (%)	35 (5.4)	47 (7.0)	39 (5.7)	43 (6.3)			
Model 1	1.00	1.32 (0.85–2.04)	0.97 (0.61–1.53)	1.03 (0.66–1.61)	0.11	1.00 (0.86–1.16)	
Model 2	1.00	1.32 (0.85–2.04)	0.97 (0.61–1.53)	1.04 (0.66–1.62)	0.77	1.00 (0.86–1.17)	
Model 3	1.00	1.22 (0.79–1.89)	0.93 (0.59–1.48)	0.84 (0.53–1.33)	0.25	0.93 (0.79–1.09)	

Model 1: adjusted for age, sex

Model 2: further adjusted for medical history (hypertension, hyperlipidemia)

Model 3: further adjusted for current smoking, alcohol consumption, estimated glomerular filtration rate, ischemic stroke subtype, randomized treatment, and baseline NIHSS score

Similarly, elevated SDF-1 levels were associated with two-year recurrent stroke and cardiovascular events only in patients with diabetes mellitus, but not those without diabetes mellitus (Supplementary Table 5).

**Fig. 1:**
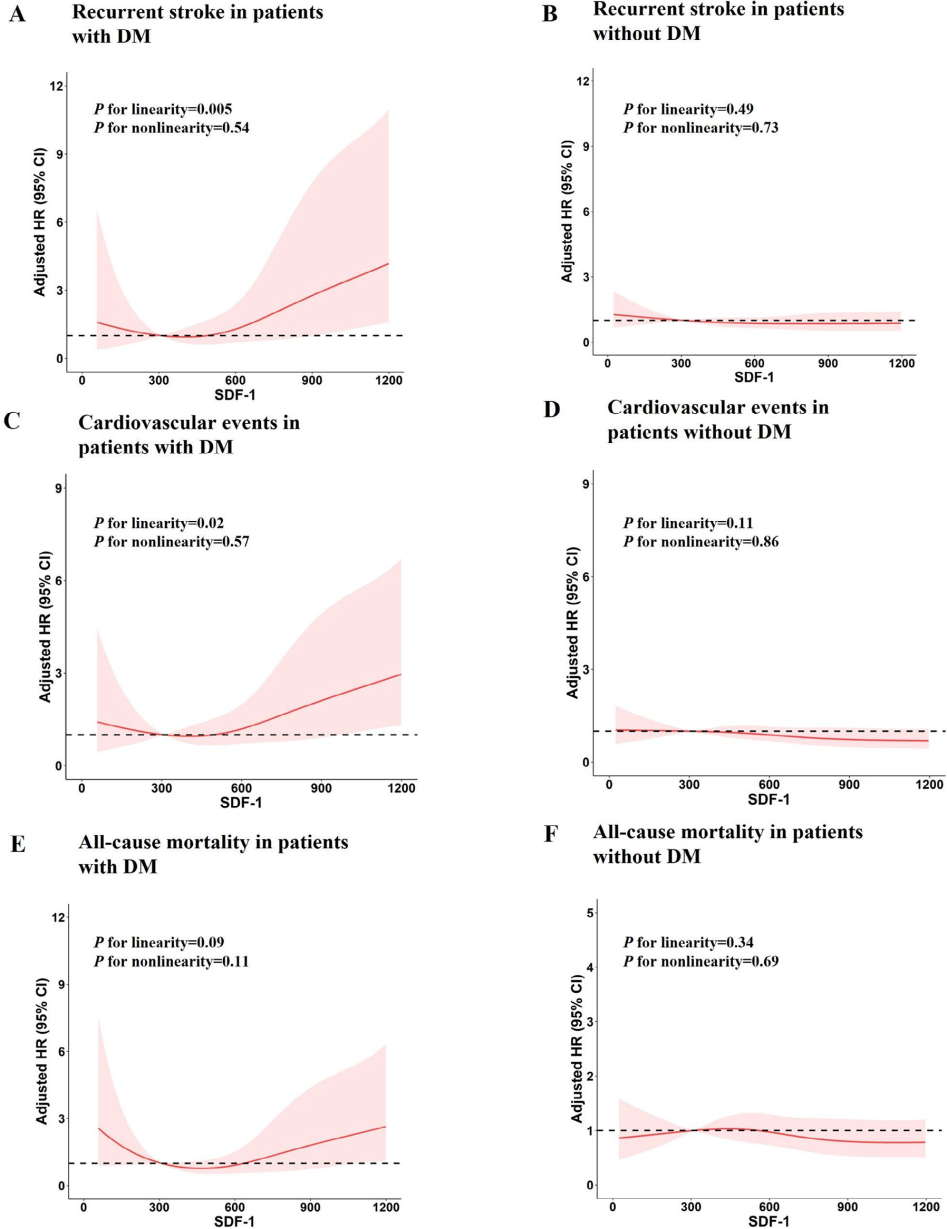
Relationships of SDF-1 with recurrent stroke, cardiovascular events and all-cause mortality in patients with ischemic stroke Hazards ratios and 95% confidence intervals derived from restricted cubic spline regression, with knots placed at the 5th, 35th, 65th, and 95th percentiles of the SDF-1 levels distribution. The reference point is the midpoint of the reference group for the categorical analysis. Hazard ratios were adjusted for the same variables as Model 3. Panel A: Recurrent Stroke at 1 year in ischemic stroke with diabetes (*P* for linearity = 0.005); Panel B: Recurrent Stroke at 1 year in ischemic stroke without diabetes (*P* for linearity = 0.49); Panel C: Cardiovascular events at 1 year in ischemic stroke with diabetes (*P* for linearity = 0.02). Panel D: Cardiovascular events at 1 year in ischemic stroke without diabetes (*P* for linearity = 0.11). Panel E: All-cause mortality at 1 year in ischemic stroke with diabetes (*P* for linearity = 0.09). Panel F: All-cause mortality at 1 year in ischemic stroke without diabetes (*P* for linearity = 0.34)

Multivariable-adjusted spline regression models showed a linear association between SDF-1 levels and recurrent stroke (Fig. 1A, P for linearity = 0.005), cardiovascular events (Fig. 1C, P for linearity = 0.02), but not all-cause mortality (Fig. 1E, P for linearity = 0.09) at one year in patients with diabetes mellitus (Fig. 1). However, no significant associations between plasma SDF-1 levels and recurrent stroke (Fig. 1B, P for linearity = 0.49), cardiovascular events (Fig. 1D, P for linearity = 0.11), all-cause mortality (Fig. 1F, P for linearity = 0.34) were detected in patients without diabetes mellitus at one year.

### Predictive performance of plasma SDF-1 levels in patients with and without diabetes mellitus

We assessed the predictive value of plasma SDF-1 beyond conventional factors for recurrent stroke, cardiovascular events, and all-cause mortality. In patients with diabetes mellitus, the Hosmer Lemeshow test indicated that model calibration was adequate after adding plasma SDF-1. Furthermore, we found that the addition of plasma SDF-1 to the basic model containing conventional risk factors also significantly improved risk reclassification for recurrent stroke (NRI: 51.77%, *P* = 0.006; IDI: 1.74%, *P* = 0.005), cardiovascular events (NRI: 42.29%, *P* = 0.008; IDI: 1.39%, *P* = 0.01), and all-cause mortality (NRI: 33.03%, *P* = 0.04; IDI: 0.69%, *P* = 0.36) at one year after stroke (Table 3). However, plasma SDF-1 did not show significance for predicting recurrent stroke, cardiovascular events, and all-cause mortality in patients without diabetes mellitus (Table 3).

**Table 3:** Performance of models with SDF-1 to predict clinical outcomes among ischemic stroke patients with different diabetes status

				Ischemic stroke patients with diabetes						Ischemic stroke patients without diabetes				
	NRI (category)				IDI		Calibration		NRI (category)		IDI		Calibration	
	Estimate (95% CI), %			*P* Value	Estimate (95% CI), %	*P* Value	χ^2^	*P* Value	Estimate (95% CI), %	*P* Value	Estimate (95% CI), %	*P* Value	χ^2^	*P* Value
**Recurrent Stroke at 1 year**														
Conventional model	Reference				Reference		10.57	0.23	Reference		Reference		7.14	0.52
Conventional model + SDF-1	51.77 (17.02–86.52)			0.006	1.74 (0.53–2.94)	0.005	7.51	0.48	5.20 (-13.09-23.50)	0.58	0.005 (-0.02-0.03)	0.75	7.96	0.44
**Cardiovascular events at 1 year**														
Conventional model	Reference				Reference		3.00	0.93	Reference		Reference		4.14	0.84
Conventional model + SDF-1	42.29 (11.73–72.84)			0.008	1.39 (0.31–2.46)	0.01	4.07	0.85	8.78 (-7.45-25.02)	0.29	0.14 (0.00-0.27)	0.05	8.71	0.37
**All-cause mortality at 1 year**														
Conventional model	Reference				Reference		18.82	0.02	Reference		Reference		9.26	0.32
Conventional model + SDF-1	33.03 (1.26–64.80)			0.04	0.69 (-0.78-2.16)	0.36	13.22	0.10	6.46 (-9.31-22.24)	0.42	0.05 (-0.11-0.21)	0.51	8.27	0.41

Abbreviations: SDF-1, stromal cell-derived factor-1; CI, confidence interval; IDI, integrated discrimination improvement; NRI, net reclassification index

The conventional model included age, sex, current smoking, alcohol consumption, estimated glomerular filtration rate, baseline NIHSS score, medical history (hypertension, hyperlipidemia), ischemic stroke subtype, and randomized treatment

## Discussion

This prospective analysis of ischemic stroke patients from the CATIS trial dataset demonstrates that higher plasma SDF-1 levels were not associated with recurrent stroke and cardiovascular events after ischemic stroke. However, we found a significant modifying role of diabetes mellitus on the association between increased plasma SDF-1 levels and poor prognosis after ischemic stroke. Our findings indicated that higher plasma SDF-1 levels were associated with increased risks of recurrent stroke and cardiovascular events within one year after ischemic stroke only in the patients with diabetes mellitus. However, no evidence of an association between plasma SDF-1 levels and clinical outcomes was found after ischemic stroke in those without diabetes mellitus.

Previous experimental studies had reported that SDF-1 activated various biological signaling pathways including atherosclerosis and inflammatory response by binding to its receptor [1, 22–24]. Several population studies indicated elevated serum SDF-1 levels were significantly associated with an increased risk of myocardial infarction and coronary artery disease [4–6, 25]. Data from the Chronic Renal Insufficiency Cohort (CRIC) Study of 3687 chronic kidney disease patients found that higher plasma SDF-1 was associated with the hazard of incident myocardial infarction and death [6]. Regarding ischemic stroke, most studies suggested that elevated serum SDF-1 was related to poor functional outcomes [8, 9, 26]. For example, a study of 304 acute ischemic stroke patients indicated that having a high level of SDF-1 (≥ 12.4 ng/mL) was an independent predictor of poor functional outcome and mortality at six months after stroke [8]. To date, one study has examined the association of SDF-1 levels with recurrent stroke. This study involved 248 patients with first-ever ischemic stroke and found an association between higher baseline serum SDF-1 levels and the risk of recurrent stroke within one year [11]. The present analysis with more than 3000 acute ischemic stroke patients showed that having elevated SDF-1 levels at baseline was not associated with an increased risk of recurrent stroke as well as cardiovascular events after ischemic stroke. The discrepant prognostic effect of SDF-1 on recurrent stroke in patients with acute ischemic stroke may largely be attributed to variations in sample size and baseline characteristics.

A growing body of evidence suggested that increased SDF-1 was strongly related to diabetes mellitus and its complications. A study of 200 patients with diabetes mellitus found that the level of SDF-1 is higher than healthy controls [12]. Several basic and clinical studies indicated that elevated level of SDF-1 was reported to contribute to diabetes complications by promoting the expression of inflammatory cytokines and immune-related disorders exacerbating insulin resistance [13–15, 27]. Moreover, inhibited expression of SDF-1 was reported to have the effect of reversing diabetic complications [28]. Additionally, a recent experimental study demonstrated that SDF-1 impaired cardiac contractility in aged, obese, and diabetic mice, but not in mice without diabetes [29]. Considering the existing evidence, we hypothesize that diabetes mellitus may modify the prognostic value of SDF-1 in ischemic stroke patients. The present study found an interaction between plasma SDF-1 levels and a history of diabetes mellitus on recurrent stroke, cardiovascular events, and all-cause mortality. In patients with diabetes mellitus, baseline plasma SDF-1 levels were significantly associated with an increased risk of recurrent stroke and cardiovascular events at one year. Moreover, the predictive effect of SDF-1 and clinical outcomes were improved after the addition of plasma SDF-1 to the basic model containing conventional risk factors. However, no associations between plasma SDF-1 levels and clinical outcomes in those without diabetes mellitus were found. Additionally, our findings revealed that having higher plasma SDF-1 levels were associated with an increased risk of recurrent stroke and cardiovascular events at two years in patients with diabetes mellitus, although this association was not as strong as observed for outcomes at one year. The underlying reason for the predictive effect of plasma SDF-1 on outcomes at two years being less robust than at one year remains unclear and needs further studies to confirm.

Our study has important clinical implications for firstly demonstrating that the status of diabetes mellitus affects the prognostic effect of plasma SDF-1 on outcomes after acute stroke. Our findings provide evidence that inflammation by diabetes mellitus may be the underlying mechanize contributing to diabetic associated cardiovascular events and help to better understand the pathogenesis of stroke progression in patients with diabetes mellitus. The findings suggested that SDF-1 could be a potential therapeutic target in acute stroke patients with diabetes mellitus in future studies and the use of SDF-1 inhibitors in medical treatment shows promise as a potential therapeutic intervention for secondary prevention following an ischemic stroke with diabetes mellitus.

The exact mechanisms underlying the relationship between higher plasma SDF-1 levels and increased risk of recurrent stroke and cardiovascular events in acute ischemic stroke patients with diabetes mellitus are unclear. Several hypotheses have been proposed. First, a growing body of evidence suggested that increased plasma SDF-1 levels can promote insulin resistance and diabetic kidney disease [12–14, 27], which were reported to be significantly associated with recurrent stroke and poor functional outcomes after ischemic stroke [30, 31]. Second, increased SDF-1 through mediate Ca^2+^ flux affects cardiac contractility in diabetes mice [29], which is associated with heart failure, cardiovascular events, and recurrent stroke.

The strengths of these analyses included the large sample size from the multicenter of the CATIS trial with standardized protocols and strict quality control procedures in baseline data collection and outcome assessment. Moreover, multiple established cardiovascular risk factors were collected and controlled in the multivariable models. However, there are also several limitations. First, the present study is derived from the CATIS trial, thus selection bias might be unavoidable. The patients with BP ≥ 220/120 mmHg or other serious conditions such as severe heart failure, acute myocardial infarction or unstable angina, atrial fibrillation, aortic dissection, or resistant hypertension at admission were excluded, so these findings cannot be generalized to these patients. Second, the possibility of residual confounding cannot be fully eliminated in an observational study, although several important potential confounders have been controlled for in multivariable-adjusted models. Third, plasma SDF-1 levels were measured only at the acute stage, and we could not evaluate the dynamic change of SDF-1 on the clinical outcomes after acute ischemic stroke.

In conclusion, we found a modifying role of diabetes mellitus on the association between baseline plasma SDF-1 levels and clinical outcomes. Elevated plasma SDF-1 was significantly associated with an increased risk of recurrent stroke and cardiovascular events at one year only in ischemic stroke patients with diabetes mellitus, but not in those without diabetes mellitus. Future studies from other populations are needed to verify our findings and clarify the potential mechanisms.

### Electronic supplementary material

Below is the link to the electronic supplementary material.

Supplementary Material 1

## Data Availability

The datasets used and analyzed in the current study are available from the corresponding author upon reasonable request.

## Nonstandard Abbreviations and Acronyms

BP: blood pressure
BMI: body mass index
CATIS: China Antihypertensive Trial in Acute Ischemic Stroke
CIs: confidence intervals
CXCL12: CXC chemokine ligand-12
CXCR4/7: CXC chemokine receptor 4/7
HRs: Hazard ratios
IDI: integrated discrimination improvement
NRI: net reclassification improvement
NIHSS: National Institutes of Health Stroke Scale
SDF-1: stromal cell-derived factor-1
