# Supplementary material for: Prognostic significance of plasma SDF-1 in acute ischemic stroke patients with diabetes mellitus: the CATIS trial
Source: Cardiovasc Diabetol. 2023 Oct 10;22:274. doi: 10.1186/s12933-023-01996-0 (PMC10566135; doi:10.1186/s12933-023-01996-0)
Supplement: Supplementary file 1 — Supplementary Material 1 [file 12933_2023_1996_MOESM1_ESM.docx]

**ONLINE-ONLY SUPPLEMENT**

**Prognostic significance of plasma SDF-1 in acute ischemic stroke patients with diabetes mellitus: the CATIS trial**

**Shoujiang You, et al.**

**Supplementary Figure 1. Patient flow chart.**

**Supplementary Table 1. Baseline characteristics of participants of Ischemic stroke patients with diabetes and without diabetes.**

**Supplementary Table 2. The events in patients with and without diabetes patients.**

**Supplementary Table 3. Associations of plasma SDF-1 with clinical outcomes at 1 year and 2 years in the whole population.**

**Supplementary Table 4. Competing risk analyses of the association of SDF-1 with 1-year clinical outcomes after ischemic stroke.**

**Supplementary Table 5. Associations between SDF-1 quartiles and stroke outcomes according to diabetes status at 2 years.**

**Supplementary Figure 1. Patient flow chart.**


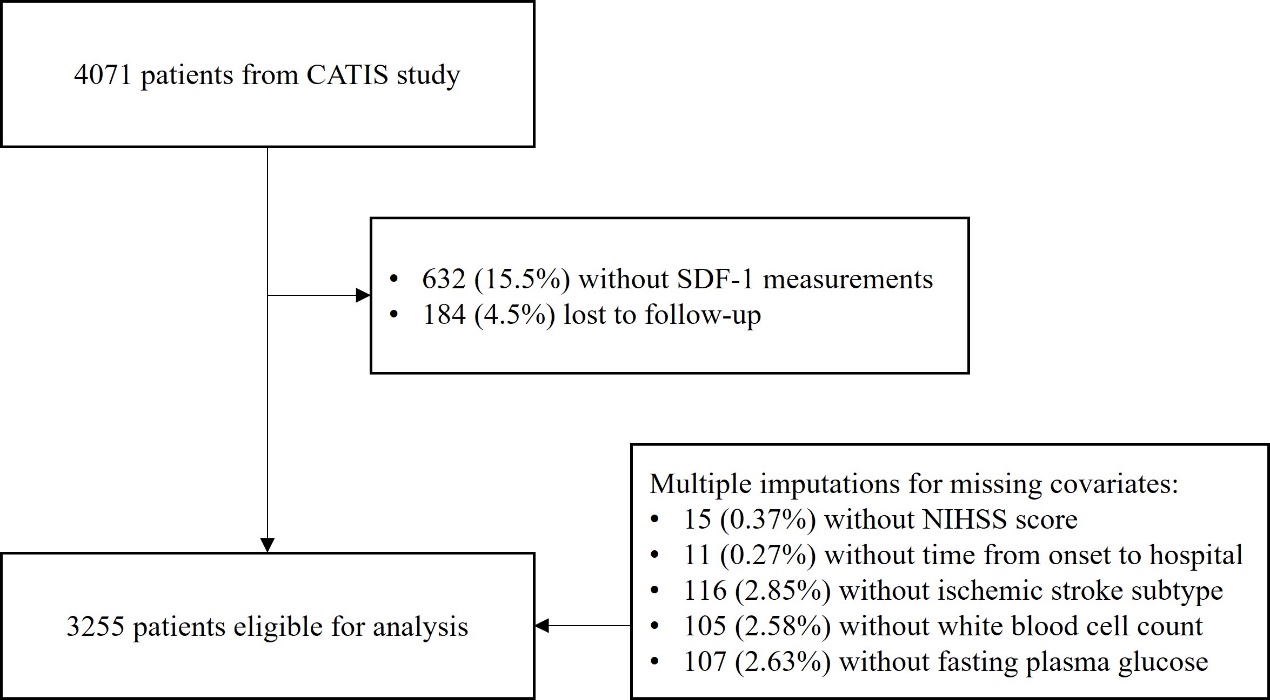


Abbreviations: CATIS, China Antihypertensive Trial in Acute Ischemic Stroke; SDF-1, stromal cell-derived factor-1; NIHSS, National Institute of Health Stroke Scale.

**Supplementary Table 1. Baseline characteristics of participants of Ischemic stroke patients with diabetes and without diabetes.**

| Characteristics | **Ischemic stroke patients with diabetes**  **(n=567)** | **Ischemic stroke patients without diabetes**  **(n=2688)** | ***P* value** |
| --- | --- | --- | --- |
| **Demographics** |  |  |  |
| Age (years) | 61.8 ± 9.6 | 61.9 ± 11.1 | 0.79 |
| Male | 338 (60.0) | 1765 (65.7) | 0.006 |
| Current cigarette smoking | 165 (29.1) | 1034 (38.5) | <.0001 |
| Current alcohol drinking | 139 (24.5) | 882 (32.8) | 0.0001 |
| **Clinical features** |  |  |  |
| SBP, mm Hg | 165.4 ±17.0 | 166.5 ± 16.7 | 0.18 |
| DBP, mm Hg | 95.1 ± 10.9 | 97.0 ± 11.1 | 0.0002 |
| WBC, 10^9^/L | 7.7 ± 4.2 | 8.1 ± 21.7 | 0.71 |
| TC, mmol/L | 5.1 (4.3-5.9) | 4.9 (4.3-5.7) | 0.003 |
| TG, mmol/L | 1.8 (.2-2.6) | 1.4 (1.0-2.1) | <.0001 |
| LDL-C, mmol/L | 3.0 (2.3-3.6) | 2.8 (2.3-3.5) | 0.007 |
| HDL-C, mmol/L | 1.0 (0.8-1.1) | 1.3 (1.0-1.5) | <.0001 |
| Time from onset to hospitalization | 11.0 (4.5-24.0) | 10.0 (4.5-24.0) | 0.60 |
| FPG, mmol/L | 9.3 ± 3.7 | 6.1 ± 2.1 | <.0001 |
| eGFR, ml/min/1.73 m2 | 105.7 (93.6-115.7) | 104.8 (93.3-114.6) | 0.38 |
| Baseline NIHSS score | 4.0 (3.0-8.0) | 4.0 (2.0-8.0) | 0.60 |
| **Medical history** |  |  |  |
| Hypertension | 486 (85.7) | 2069 (77.0) | <.0001 |
| Hyperlipidemia | 90 (15.9) | 144 (5.4) | <.0001 |
| Coronary heart disease | 96 (16.9) | 238 (8.9) | <.0001 |
| Family history of stroke | 120 (21.2) | 482 (17.9) | 0.07 |
| **Prior use of medications** |  |  |  |
| Antihypertensive | 339 (60.0) | 1238 (46.1) | <.0001 |
| Lipid–lowing | 39 (6.9) | 68 (2.5) | <.0001 |
| **Ischemic stroke subtype** |  |  | 0.75 |
| Thrombotic | 444 (78.3) | 2080 (77.4) | 0.63 |
| Embolic | 26 (4.6) | 132 (4.9) | 0.74 |
| Lacunar | 106 (18.7) | 541 (20.1) | 0.44 |
| Randomized treatment | 289 (51.0) | 1348 (50.2) | 0.72 |
| SDF-1 | 624.5 ± 314.6 | 673.7 ± 336.7 | 0.001 |

Continuous variables are expressed as mean ± standard deviation or median (interquartile range).

Categorical variables are expressed as frequency (percentage).

Abbreviations: SDF-1, stromal cell-derived factor-1; FPG, fasting plasma glucose; NIHSS, National Institute of Health Stroke Scale; SBP, systolic blood pressure; DBP, diastolic blood pressure; TG, triglycerides; TC, total cholesterol; LDL-C, low density lipoprotein cholesterol; HDL-C, high density lipoprotein cholesterol; WBC, white blood cell; eGFR, estimated glomerular filtration rate.

**Supplementary Table 2. The events in patients with and without diabetes patients.**

|  | **Total patients** | **With diabetes** | **Without diabetes** | **P value** |
| --- | --- | --- | --- | --- |
| **Outcomes at 1 year** |  |  |  |  |
| Recurrent stroke | 150 (4.6%) | 30 (5.3%) | 120 (4.5%) | 0.39 |
| Cardiovascular events | 196 (6.0%) | 42 (7.4%) | 154 (5.7%) | 0.13 |
| All-cause mortality | 204 (6.3%) | 40 (7.1%) | 164 (6.1%) | 0.39 |
| **Outcomes at 2 years** | |  |  |  |
| Recurrent stroke | 258 (7.9%) | 50 (8.8%) | 208 (7.7%) | 0.39 |
| Cardiovascular events | 318 (9.8%) | 65 (11.5%) | 253 (9.4%) | 0.13 |
| All-cause mortality | 281 (8.1%) | 55 (9.7%) | 226 (8.4%) | 0.32 |

**Supplementary Table 3. Associations of plasma SDF-1 with clinical outcomes at 1 year and 2 years in the whole population**

|  | **SDF-1,** **pg/mL** | | | |  | **Each 1-SD increase** | |
| --- | --- | --- | --- | --- | --- | --- | --- |
|  | **Q1** | **Q2** | **Q3** | **Q4** | ***P* trend** |  |  |
| Median | 309.0 | 540.0 | 735.2 | 1025.0 |  |  |  |
| **Recurrent stroke at 1 year** | |  |  |  |  |  |  |
| Events, n (%) | 37 (4.6) | 35 (4.3) | 33 (4.1) | 45 (5.5) |  |  |  |
| Model 1 | 1.00 | 0.95 (0.60-1.51) | 0.86 (0.54-1.38) | 1.18 (0.76-1.82) | 0.48 | 1.03 (0.88-1.21) |  |
| Model 2 | 1.00 | 0.95 (0.60-1.51) | 0.87 (0.55-1.40) | 1.18 (0.76-1.83) | 0.47 | 1.03 (0.88-1.21) |  |
| Model 3 | 1.00 | 0.94 (0.59-1.50) | 0.87 (0.55-1.40) | 1.22 (0.79-1.90) | 0.38 | 1.04 (0.89-1.23) |  |
| **Cardiovascular events at 1 year** | |  |  |  |  |  |  |
| Events, n (%) | 50 (6.2) | 47 (5.8) | 50 (6.2) | 49 (6.0) |  |  |  |
| Model 1 | 1.00 | 0.94 (0.63-1.40) | 0.96 (0.65-1.42) | 0.93 (0.63-1.39) | 0.76 | 0.97 (0.84-1.12) |  |
| Model 2 | 1.00 | 0.94 (0.63-1.40) | 0.96 (0.65-1.43) | 0.94 (0.63-1.39) | 0.77 | 0.97 (0.84-1.12) |  |
| Model 3 | 1.00 | 0.92 (0.62-1.37) | 0.96 (0.64-1.42) | 0.93 (0.62-1.39) | 0.78 | 0.97 (0.84-1.12) |  |
| **All-cause mortality at 1 year** | |  |  |  |  |  |  |
| Events, n (%) | 44 (5.4) | 55 (6.7) | 48 (5.9) | 57 (7.0) |  |  |  |
| Model 1 | 1.00 | 1.27 (0.85-1.88) | 1.01 (0.67-1.52) | 1.16 (0.78-1.72) | 0.70 | 1.04 (0.90-1.19) |  |
| Model 2 | 1.00 | 1.26 (0.85-1.88) | 1.01 (0.67-1.52) | 1.17 (0.79-1.73) | 0.68 | 1.04 (0.91-1.19) |  |
| Model 3 | 1.00 | 1.15 (0.77-1.71) | 1.00 (0.66-1.50) | 0.98 (0.66-1.47) | 0.74 | 0.98 (0.85-1.13) |  |
| **Recurrent stroke at 2 years** | |  |  |  |  |  |  |
| Events, n (%) | 63 (7.8) | 69 (8.5) | 56 (6.9) | 70 (8.6) |  |  |  |
| Model 1 | 1.00 | 1.10 (0.78-1.55) | 0.87 (0.61-1.25) | 1.10 (0.78-1.55) | 0.83 | 0.97 (0.85-1.10) |  |
| Model 2 | 1.00 | 1.11 (0.79-1.56) | 0.88 (0.61-1.26) | 1.10 (0.78-1.55) | 0.80 | 0.97 (0.85-1.09) |  |
| Model 3 | 1.00 | 1.11 (0.79-1.56) | 0.88 (0.62-1.27) | 1.15 (0.82-1.63) | 0.62 | 0.98 (0.86-1.11) |  |
| **Cardiovascular events at 2 years** | |  |  |  |  |  |  |
| Events, n (%) | 80 (9.9) | 86 (10.5) | 77 (9.5) | 75 (9.2) |  |  |  |
| Model 1 | 1.00 | 1.08 (0.79-1.46) | 0.94 (0.69-1.29) | 0.92 (0.67-1.27) | 0.48 | 0.92 (0.82-1.04) |  |
| Model 2 | 1.00 | 1.08 (0.79-1.46) | 0.95 (0.69-1.29) | 0.93 (0.67-1.27) | 0.77 | 0.92 (0.82-1.04) |  |
| Model 3 | 1.00 | 1.08 (0.79-1.45) | 0.95 (0.69-1.30) | 0.94 (0.68-1.29) | 0.55 | 0.93 (0.83-1.04) |  |
| **All-cause mortality at 2 years** | |  |  |  |  |  |  |
| Events, n (%) | 55 (6.8) | 77 (9.4) | 66 (8.1) | 83 (10.2) |  |  |  |
| Model 1 | 1.00 | 1.45 (1.03-2.05) | 1.09 (0.76-1.56) | 1.37 (0.97-1.92) | 0.22 | 1.06 (0.95-1.19) |  |
| Model 2 | 1.00 | 1.45 (1.02-2.05) | 1.09 (0.76-1.56) | 1.37 (0.97-1.92) | 0.22 | 1.06 (0.95-1.19) |  |
| Model 3 | 1.00 | 1.33 (0.94-1.89) | 1.08 (0.76-1.55) | 1.20 (0.85-1.70) | 0.56 | 1.02 (0.91-1.15) |  |

Abbreviations: SD, standard deviation;

Model 1: adjusted for age, sex.

Model 2: adjusted for age, sex, medical history (hypertension, hyperlipidemia).

Model 3: further adjusted for current smoking, alcohol consumption, estimated glomerular filtration rate, ischemic stroke subtype, randomized treatment, and baseline NIHSS score.

**Supplementary Table 4. Competing risk analyses of the association of SDF-1 with 1-year clinical outcomes after ischemic stroke.**

|  |  |  | SDF-1 |  |  | **Continuous** |
| --- | --- | --- | --- | --- | --- | --- |
|  | **Q1** | **Q2** | **Q3** | **Q4** | ***P* trend** | **(per SD increase)** |
| **Ischemic stroke patients with diabetes** | | | |  |  |  |
| **Recurrent Stroke at 1 year** | |  |  |  |  |  |
| Events (%) | 4 (2.4) | 6 (4.1) | 8 (6.4) | 12 (9.5) |  |  |
| Model 1 | 1.00 (ref) | 1.72 (0.48-6.11) | 2.58 (0.78-8.54) | 3.92 (1.27-12.12) | 0.007 | 1.67 (0.16-2.41) |
| Model 2 | 1.00 (ref) | 1.79 (0.50-6.35) | 2.65 (0.80-8.80) | 4.04 (1.31-12.48) | 0.006 | 1.65 (1.15-2.35) |
| Model 3 | 1.00 (ref) | 1.73 (0.49-6.14) | 2.76 (0.81-9.43) | 4.00 (1.33-12.01) | 0.004 | 1.65 (1.16-2.34) |
| **Cardiovascular events at 1 year** | |  |  |  |  |  |
| Events (%) | 7 (4.2) | 9 (6.1) | 12 (9.5) | 14 (11.0) |  |  |
| Model 1 | 1.00 (ref) | 1.76 (0.50-6.27) | 2.61 (0.79-8.65) | 4.01 (1.30-12.40) | 0.006 | 1.66 (1.16-2.39) |
| Model 2 | 1.00 (ref) | 1.81 (0.51-6.45) | 2.65 (0.80-8.81) | 4.16 (1.35-12.85) | 0.005 | 1.64 (1.16-2.33) |
| Model 3 | 1.00 (ref) | 1.76 (0.50-6.24) | 2.73 (0.80-9.30) | 4.06 (1.36-12.15) | 0.004 | 1.64 (1.16-2.31) |
| **Ischemic stroke patients without diabetes** | | | |  |  |  |
| **Recurrent Stroke at 1 year** | |  |  |  |  |  |
| Events (%) | 33 (5.1) | 29 (4.3) | 25 (3.6) | 33 (4.8) |  |  |
| Model 1 | 1.00 (ref) | 0.85 (0.51-1.39) | 0.69 (0.41-1.17) | 0.90 (0.55-1.47) | 0.63 | 0.92 (0.76-1.12) |
| Model 2 | 1.00 (ref) | 0.85 (0.51-1.39) | 0.70 (0.42-1.19) | 0.91 (0.55-1.48) | 0.65 | 0.92 (0.76-1.12) |
| Model 3 | 1.00 (ref) | 0.84 (0.51-1.39) | 0.71 (0.42-1.20) | 0.96 (0.59-1.57) | 0.81 | 0.94 (0.77-1.14) |
| **Cardiovascular events at 1 year** | |  |  |  |  |  |
| Events (%) | 43 (6.7) | 38 (5.7) | 38 (5.5) | 35 (5.1) |  |  |
| Model 1 | 1.00 (ref) | 0.86 (0.52-1.41) | 0.69 (0.41-1.16) | 0.90 (0.55-1.47) | 0.62 | 0.92 (0.76-1.12) |
| Model 2 | 1.00 (ref) | 0.86 (0.52-1.41) | 0.70 (0.41-1.18) | 0.91 (0.56-1.48) | 0.64 | 0.92 (0.76-1.12) |
| Model 3 | 1.00 (ref) | 0.86 (0.52-1.41) | 0.70 (0.41-1.19) | 0.95 (0.58-1.56) | 0.77 | 0.94 (0.77-1.14) |

Model 1: adjusted for age, sex.

Model 2: further adjusted for medical history (hypertension, hyperlipidemia).

Model 3: further adjusted for current smoking, alcohol consumption, estimated glomerular filtration rate, ischemic stroke subtype, and baseline NIHSS score randomized treatment.

**Supplementary Table 5. Associations between SDF-1 quartiles and stroke outcomes according to diabetes status at 2 years.**

|  | **SDF-1,** **pg/mL** | | | |  | **Each 1-SD increase** | |
| --- | --- | --- | --- | --- | --- | --- | --- |
|  | **Q1** | **Q2** | **Q3** | **Q4** | ***P* trend** |  |  |
| **Ischemic stroke patients with diabetes** | | | | |  |  |  |
| **Recurrent stroke at 2 years** | |  |  |  |  |  |  |
| Events, n (%) | 8 (4.8) | 16 (10.8) | 11 (8.7) | 15 (11.8) |  |  |  |
| Model 1 | 1.00 | 2.32 (0.99-5.43) | 1.87 (0.75-4.67) | 2.61 (1.11-6.17) | 0.05 | 1.29 (0.98-1.71) |  |
| Model 2 | 1.00 | 2.38 (1.02-5.57) | 1.89 (0.76-4.74) | 2.68 (1.13-6.34) | 0.05 | 1.29 (0.98-1.70) |  |
| Model 3 | 1.00 | 2.44 (1.04-5.71) | 1.88 (0.75-4.72) | 2.81 (1.18-6.70) | 0.04 | 1.32 (0.99-1.75) |  |
| **Cardiovascular events at 2 years** | |  |  |  |  |  |  |
| Events, n (%) | 12 (7.2) | 20 (13.5) | 15 (11.9) | 18 (14.2) |  |  |  |
| Model 1 | 1.00 | 1.95 (0.95-3.99) | 1.67 (0.78-3.58) | 2.06 (0.99-4.28) | 0.09 | 1.20 (0.94-1.55) |  |
| Model 2 | 1.00 | 1.99 (0.97-4.06) | 1.69 (0.79-3.62) | 2.10 (1.01-4.36) | 0.02 | 1.21 (0.94-1.55) |  |
| Model 3 | 1.00 | 2.03 (0.99-4.15) | 1.67 (0.77-3.60) | 2.25 (1.07-4.73) | 0.06 | 1.25 (0.97-1.62) |  |
| **All-cause mortality at 2 years** | |  |  |  |  |  |  |
| Events, n (%) | 12 (7.2) | 12 (8.1) | 13 (10.3) | 18 (14.2) |  |  |  |
| Model 1 | 1.00 | 1.27 (0.56-2.87) | 1.50 (0.67-3.35) | 2.06 (0.97-4.37) | 0.05 | 1.26 (0.96-1.66) |  |
| Model 2 | 1.00 | 1.24 (0.55-2.82) | 1.50 (0.67-3.33) | 2.02 (0.95-4.29) | 0.05 | 1.25 (0.95-1.66) |  |
| Model 3 | 1.00 | 1.13 (0.50-2.58) | 1.52 (0.67-3.44) | 2.18 (1.00-4.75) | 0.03 | 1.34 (0.99-1.80) |  |
| **Ischemic stroke patients without diabetes** | | | | |  |  |  |
| **Recurrent stroke at 2 years** | |  |  |  |  |  |  |
| Events, n (%) | 55 (8.5) | 53 (7.9) | 45 (6.6) | 55 (8.0) |  |  |  |
| Model 1 | 1.00 | 0.94 (0.64-1.37) | 0.75 (0.51-1.11) | 0.92 (0.63-1.34) | 0.53 | 0.91 (0.79-1.05) |  |
| Model 2 | 1.00 | 0.94 (0.64-1.37) | 0.76 (0.51-1.13) | 0.93 (0.64-1.35) | 0.56 | 0.91 (0.79-1.05) |  |
| Model 3 | 1.00 | 0.93 (0.64-1.36) | 0.77 (0.52-1.14) | 0.97 (0.67-1.42) | 0.74 | 0.92 (0.80-1.07) |  |
| **Cardiovascular events at 2 years** | |  |  |  |  |  |  |
| Events, n (%) | 68 (10.5) | 66 (9.9) | 62 (9.0) | 57 (8.3) |  |  |  |
| Model 1 | 1.00 | 0.91 (0.65-1.28) | 0.83 (0.59-1.17) | 0.76 (0.54-1.09) | 0.11 | 0.87 (0.76-0.99) |  |
| Model 2 | 1.00 | 0.91 (0.65-1.29) | 0.84 (0.59-1.18) | 0.77 (0.54-1.09) | 0.16 | 0.87 (0.76-0.99) |  |
| Model 3 | 1.00 | 0.90 (0.64-1.26) | 0.83 (0.59-1.18) | 0.76 (0.53-1.09) | 0.13 | 0.87 (0.76-0.99) |  |
| **All-cause mortality at 2 years** | |  |  |  |  |  |  |
| Events, n (%) | 43 (6.7) | 65 (9.7) | 53 (7.7) | 65 (9.5) |  |  |  |
| Model 1 | 1.00 | 1.49 (1.02-2.19) | 1.04 (0.69-1.55) | 1.25 (0.85-1.84) | 0.21 | 1.03 (0.91-1.17) |  |
| Model 2 | 1.00 | 1.49 (1.01-2.19) | 1.04 (0.69-1.56) | 1.25 (0.85-1.84) | 0.62 | 1.03 (0.91-1.17) |  |
| Model 3 | 1.00 | 1.38 (0.94-2.04) | 1.01 (0.67-1.51) | 1.06 (0.72-1.57) | 0.75 | 0.97 (0.85-1.11) |  |

Model 1: adjusted for age, sex.

Model 2: further adjusted for medical history (hypertension, hyperlipidemia).

Model 3: further adjusted for current smoking, alcohol consumption, estimated glomerular filtration rate, ischemic stroke subtype, randomized treatment, and baseline NIHSS score.

*P* _interaction_: Recurrent Stroke at 2 years: 0.05; Cardiovascular events at 2 years: 0.02; All-cause mortality at 2 years: 0.06.
